# Unique duck rearing practice in irrigated rice paddy fields driving recurrent H5N1 avian influenza outbreaks in two districts of Kerala, India

**DOI:** 10.1017/S0950268824001882

**Published:** 2025-01-07

**Authors:** Mohammed Mudassar Chanda, Sathish Bhadravati Shivachandra, Adhiraj Mishra, Previn Punnoose, Shaji Panikkassery, Sanjay Devarajan Potti, Vysakh Mohan, Awadhesh Prajapati, Revanaiah Yogisharadhya, Divakar Hemadri, Baldev Raj Gulati, Chakradhar Tosh

**Affiliations:** 1 ICAR-National Institute of Veterinary Epidemiology and Disease Informatics (NIVEDI), Bengaluru-560119, Karnataka, India; 2 Department of Animal Husbandry and Dairying, Ministry of Fisheries, Animal Husbandry and Dairying, New Delhi, India; 3 Kerala State Animal Husbandry Department, Government of Kerala, Thiruvananthapuram-695033, Kerala, India; 4 ICAR- Krishi Vigyan Kendra, ICAR RC for NEH Region, Hailakhandi-788155, Assam, India; 5 ICAR-National Institute of High Security Animal Diseases, Bhopal-462022, Madhya Pradesh, India

**Keywords:** Recurrent HPAI outbreaks, Kerala, duck rearing, rice irrigated areas, wetlands, risk factors, Bayesian network modeling, SPDE models

## Abstract

Highly pathogenic avian influenza (HPAI) outbreaks have repeatedly occurred in two districts of Kerala state, India, over the last few years. The outbreaks in the wetland areas coincided with the arrival of migratory birds. At the time, the factors responsible for local transmission in ducks were not known. This study aimed to identify the socio-economic factors responsible for spatial variation in the occurrence of HPAI outbreaks in the two districts using Bayesian network modelling (BNM) and Stochastic Partial Differential Equation (SPDE) model. Further, information was collected on the duck rearing practices in rice paddy fields to identify the risk factors for local – spread of the outbreaks. We found that the SPDE model without covariates explained variation in occurrence of outbreaks. The number of rice paddy fields used by the duck farmers was identified as risk factor. We concluded based on BNM and SPDE that the infected migratory birds were the source of infection for the first few duck farms in the wetland areas and subsequent transmission was driven by shifting of ducks from one rice paddy field to other fields. There is a probability of persistent and recurrent infections in the ducks and possible spill over to humans. Hence, it is important to have surveillance in ducks to prevent recurrent outbreaks in the region.

## Introduction

There are many factors responsible for the recurrence of highly pathogenic avian influenza (HPAI) outbreaks in a region. Recurrence can be due to the presence of asymptomatic infections of H5N1, H5N2, H9N1, and H9N2 in chickens with poor biosecurity measures on the farms and other factors [1]. Poor biosecurity measures have often been identified as a significant factor along with others showing that there was transmission of these viruses between farms without clinical signs [1]. It is observed that there was high prevalence of H5N1 and H9N2 viruses in apparently healthy birds and the prevalence of these viruses is more in backyard poultry [2], and more studies are required on the maintenance of these viruses in poultry and ducks which pose a serious threat of spill over in humans. The presence of influenza type A and H5 and higher incidence in ducks reared for meat purpose throughout the year without clinical disease shows the circulation of the virus in ducks and the need for viral surveillance in high-risk areas [2, 3].

Currently, 18 hemagglutinin (HA) and 11 neuraminidase (NA) subtypes in type A influenza viruses have been recognized globally [4]. However, in birds 16 HA and 9 NA subtypes have been detected, and the other two subtypes – H17N10 and H18N11 – detected in bats. Wild waterfowl and shorebirds are natural reservoirs for low pathogenicity avian influenza (LPAI) viruses [5, 6]. Infected reservoirs remain asymptomatic and can shed the virus into the environment, mainly through faeces [7]. The current global panzootic in mammals has resulted in outbreaks of H5N1 in many mammals including cattle and it has sustained mammal-to-mammal transmission [8]. The occurrence of the first outbreak in India was found to be associated with migratory birds as a source of infection and subsequent spread depends on the local factors [9, 10].

The unique geography of Kerala makes the state distinct from other states of the country. Some areas of Ernakulum, Alappuzha, and Kottayam districts of Kerala are covered by coastal wetland. Rice-duck cultivation practice in wetlands is economically and ecologically sustainable [11]. It not only provides feed to ducks, but the secretions, excreta, pecking, and predation decrease the occurrence of plant diseases, pests, and weeds in the rice paddy fields. Mainly two local breeds of ducks are raised in the state namely *Chara* and *Chembally* (*Anas platyrhynchos domesticus* Linnaeus, 1758). These breeds are unique to the state and are reared for meat and egg production purposes. In addition to these, some farmers use a broiler breed of duck such as *Vigova* (*A. platyrhynchos domesticus* Linnaeus, 1758) in an intensive system of rearing. The meat purpose ducks are reared up to 3–3.5 months (mainly males) and egg purpose ducks are reared up to 2 years. The average holding capacity of farmers ranges from 100 to 25,000 ducks. The bigger holders are the farmers who run their own private hatcheries. Small holdings are mainly for egg purpose, wherein ducks are either confined to their surroundings or let loose to their own paddy fields in their vicinity with access to the migratory birds. The first outbreak of H5N1 in Kerala was reported in 2014, and since then there have been regular reports of H5N1 or H5N8 in the state especially in the two districts, namely Alappuzha and Kottayam. It is not known what factors may be responsible for the spread of the disease in the region.

In analyses of epidemiological data and risk factor identification, a multitude of potential predictor variables must be reduced to a subset that is most strongly associated with the outbreaks. Variable selection in epidemiological studies is an important step in model building. The variables can be narrowed down based on the expertise of a researcher or chosen from many variable selection methods available. These variable selection methods are based on significant *p*-values [12], step-wise forward or backward procedures [13], information criterion such as AIC (Akaike information criterion), corrected AIC (AICc), and Bayesian Information Criteria (BIC) [14], Least Absolute Shrinkage and Selection Operator (LASSO) methods [15], least angle or penalized regression [16], and all subsets approaches [17]. In this study, we employed a Bayesian network modelling (BNM) [18] which can identify the relationship between variables and can be further used in predictive or other modelling methods. It can be considered as a multivariate regression method in a Bayesian framework. The BNM approach has been employed in epidemiological studies for risk factor identification [19] [20, 21].

Once the variables are identified as associated with the occurrence of HPAI, we need to identify the spatial pattern/spatial autocorrelation in the spread of the disease. The presence of spatial pattern or influence of covariates or both are important in driving the spatial variation in occurrence of a disease that needs to be identified. There are many spatial methods [22–25] that can be used to identify spatial pattern in the occurrence of outbreaks. The presence of spatial autocorrelation in data is difficult to fit in a BNM framework due to complex geostatistical structures and difficulty in fitting of all possible networks. Hence, BNM was used in this present study to identify the direct and indirect association between different village level variables in two districts of Kerala as the first step. The variables that were directly or indirectly associated with the HPAI outbreaks identified using BNM were then used to fit an SPDE spatial model by accounting for spatial autocorrelation.

The present study aimed to answer the following questions with respect to occurrence of HPAI in two districts of Kerala

1. What are the socio-economic factors associated with the occurrence of HPAI outbreaks?
2. Is there any spatial pattern in occurrence of HPAI outbreaks and if it can be explained by a spatial model?

## Materials and methods

### Avian influenza outbreaks data for Kerala

HPAI (H5N1 and H5N8) avian Influenza outbreak details were obtained from the World Organisation for Animal Health (WOAH) portal (https://www.woah.org/en/what-we-do/animal-health-and-welfare/disease-data-collection/world-animal-health-information-system) for two districts of Kerala state, namely Alappuzha and Kottayam, for the years 2014–2022. Further, details on the susceptible number of duck populations for all the panchayats (admin level 4) were obtained from the Kerala Animal Husbandry Department. The proportion (number of cases/number of susceptible duck population in particular panchayat) of HPAI outbreaks was calculated and used for further analysis.

*Socio-economic data:* The socio-economic variables considered were number of households, total population, literacy (literate and illiterate), total expenditure, total income, and presence of irrigation variables (lake, tank, river, water fall, other irrigation, un-irrigated, and total irrigation) were obtained from the human census data of India, 2011. There were a total of 17 variables including proportion of HPAI that was included to fit a BNM model at village level.

*BNM:* The BNM was fitted to the HPAI outbreaks in Kerala and the census variables using the *abn* package in R [26]. The main aim in BNM is to identify the relationships and interdependencies between variables. An uninformative structural prior was used, meaning that all the structures have equal chances of being selected in the final DAG, and uninformative Gaussian priors with mean zero and variance 1,000 were assumed for the parameters defining relationships between all the variables. A globally optimal DAG is then identified by a process of structure discovery or structure learning. The exact search method based on a goodness of fit criterion that was the highest log marginal likelihood score (network score) was used in this study [27]. The best DAG was identified by fitting 2*^n^* models, where *n* is the number of variables (in this case 2^17^ = 131,072 models were fitted with each parent limit). Ten parent limits were tried and the parent limit was decided based on highest likelihood score.

### Bayesian SPDE model in INLA

The semi-variogram graph illustrates the spatial autocorrelation of the measured sample points. The distance at which the graph levels off is referred to as the range. The locations within the range are considered as spatially autocorrelated. The value on the *y*-axis where the semi-variogram graph reaches its range is referred to as the sill. The nugget is the semi-variance at distance zero and is usually attributed to non-normal data. The semi-variogram was plotted using R package gstat [28].

Until recently, it was not possible to fit geostatistical correlation structures with the integrated nested Laplace approximation (INLA) approach, but this was overcome by using a Stochastic Partial Differential Equation (SPDE) approximation of the Gaussian field (GF) by the Gaussian Markov random field (GMRF) [29]. The SPDE approximation of the GF with GMRF is promising but requires pre-processing of the data to create a triangulation matrix [30]. The SPDE model in R-INLA [31] using a Delaunay triangulation approach to create mesh and was fit to the HPAI outbreaks data along with socio-economic variables identified in the BNM approach. A Delaunay triangulation is constructed using points of locations of the outbreaks and it consists of non-overlapping triangles. The triangles can be of different sizes and angles. This method of triangulation is suited for interpolation. The socio-economic variables identified in BNM as indirectly associated with proportion of HPAI outbreaks were rasterized at the panchayat level (admin level 4) and used in the SPDE model as independent variables. In Bayesian analysis, the confidence intervals of co-efficient that do not bridge zero were considered as significant.

Two models were fit, one with covariates and one without covariates. Relative performance of the two models was assessed using a deviance information criteria (DIC), with the lowest DIC score indicating the best fitting model [32].

## Model description

*Mesh construction*: Mesh was constructed with location of the HPAI outbreaks.

True prevalence of the diseases 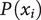 at location 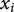 *i* = 1,…….., *n*, the number of cases 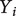 out of 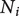 susceptible duck populations follows a Poisson distribution

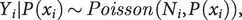




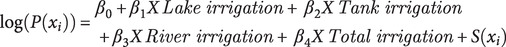

Where 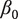 denotes the intercept, and 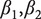 , 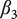 , and 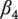 are the coefficients of lake irrigation, tank irrigation, river irrigation, and total irrigation, respectively. 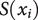 is the spatial random effect that follows a zero-mean Gaussian process with Matérn covariance function.

*Unsampled locations for making predictions*: The locations on which predictions are to be made were specified restricted to two districts.

### Questionnaire data collection from duck farmers and logistic regression model

The details of all the duck farmers in the Alappuzha and Kottayam districts were obtained from district level authorities that maintain the list and update it every time whenever there is any addition of new farmers or discontinuation of old farms. A brief questionnaire was prepared with the objective of identifying factors responsible for occurrence and spread of the disease in these two districts to collect information from duck farmers. The questionnaire included the following details: *Name of the panchayat*, *report of avian influenza during the past 3 years*, *purpose of rearing of ducks (layer, broiler, or both)*, *rearing in paddy field or other places, and number of paddy fields covered in a year* (Table S1). Once the data was collected from individual duck farmers, attack rate was calculated for different variables separately. Further, it was analysed using logistic regression models with occurrence of avian influenza as the dependent variable and other variables as independent variables using logistic regression model in R [33]. The type of the farm (layer, meat purpose, both layer and broiler) was considered as categorical variable. The information on movement of ducks in different paddy fields collected using questionnaire data and interacting with farmers was used to create illustration.

## Results

**Figure 1.**
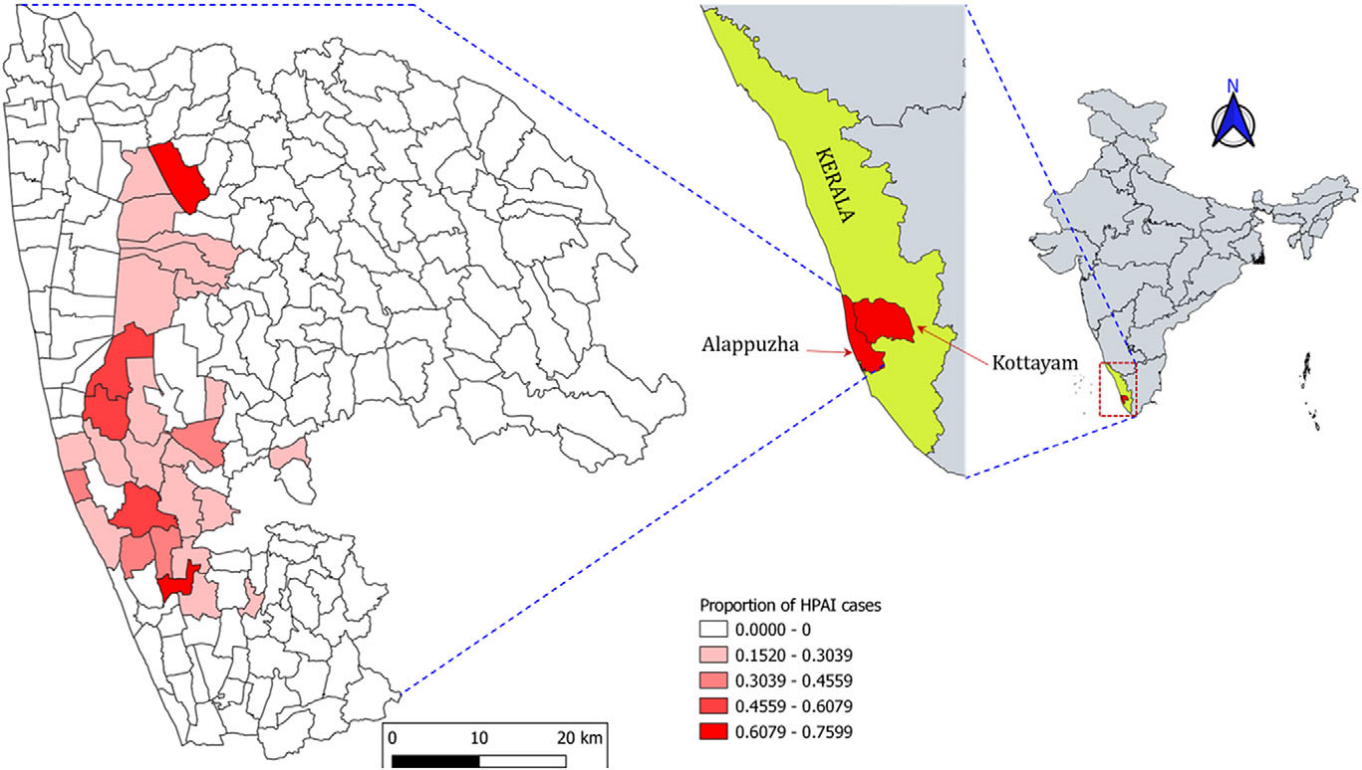
Proportion of HPAI cases (H5N1 and H5N8) in Alappuzha and Kottayam districts. Inset: Kerala state in India and two districts.

**Figure 2.**
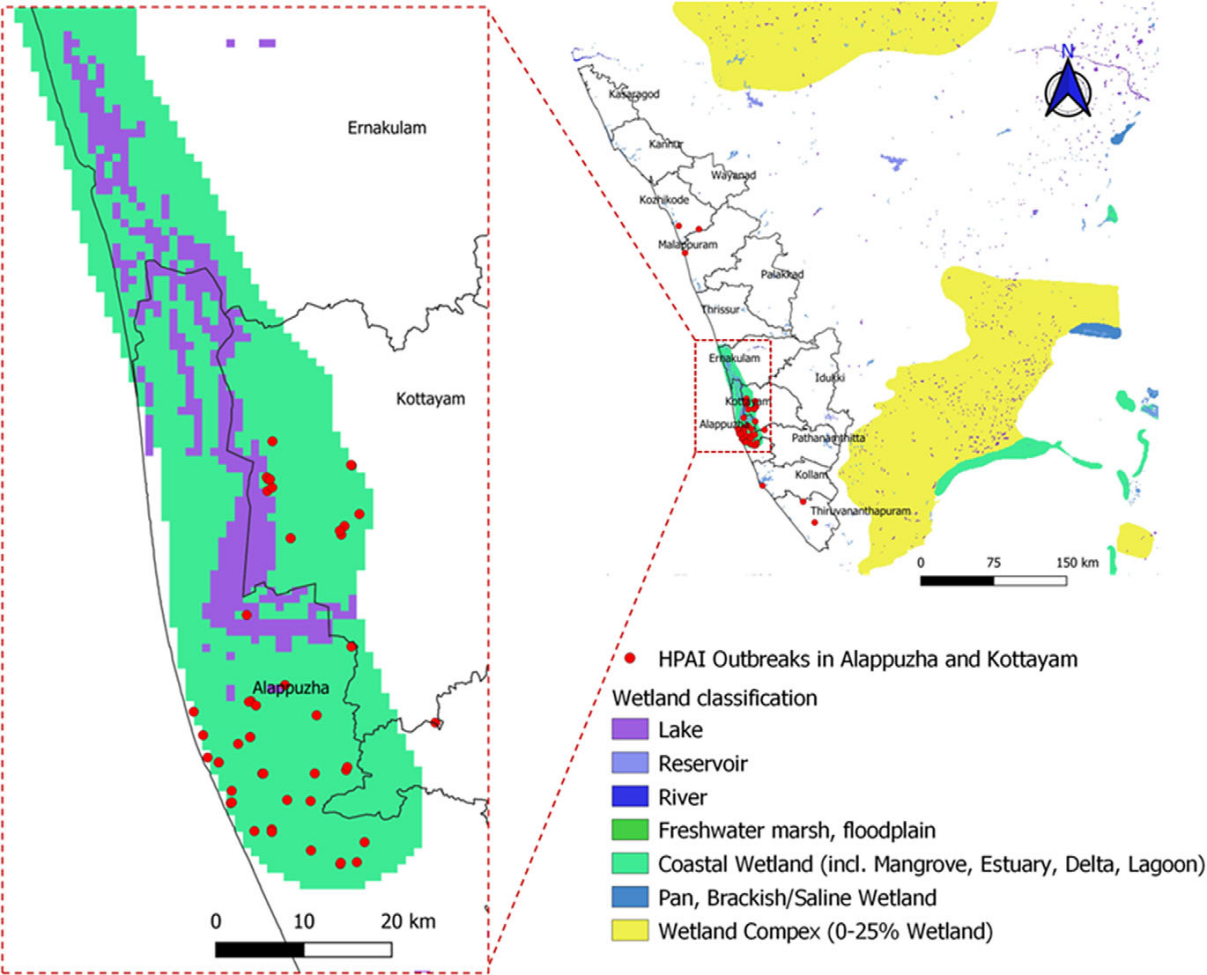
Wetland areas in Alappuzha and Kottayam districts. Majority of proportion of class coastal wetland (including Mangroove, Estuary, Delta, and Lagoon) is in Alappuzha and Kottayam districts.

The proportion of avian influenza outbreaks in panchayats of two districts in Kerala is shown in Figure 1. The distribution of wetland areas and HPAI outbreaks (Figure 2) shows that the wetland classification coastal wetland (including mangrove, estuary delta, and lagoon) is mainly distributed in Alappuzha and Kottayam districts of Kerala.

### BNM modelling results

**Figure 3.**
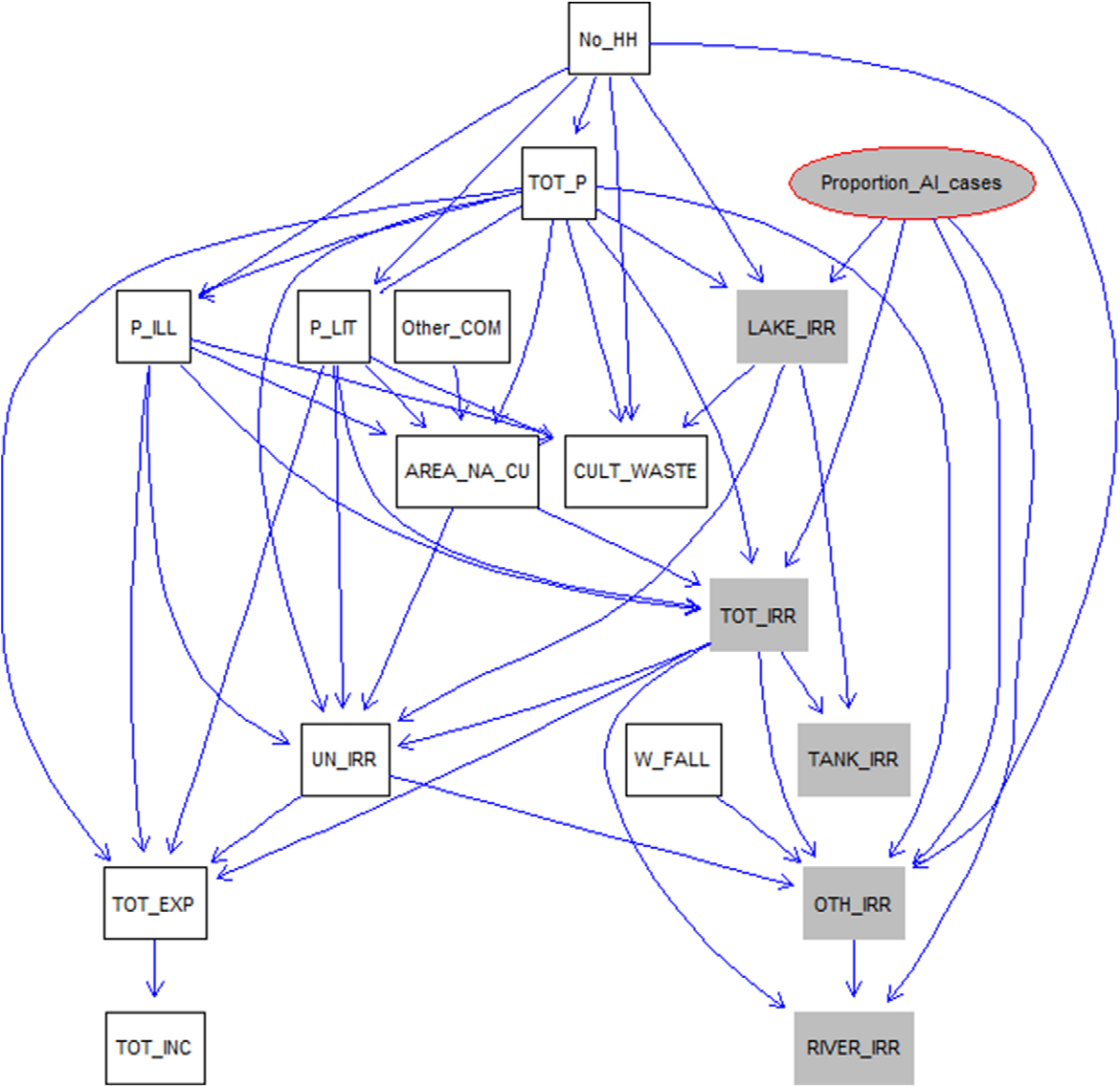
Network graph of socio-economic variables and proportion of HPAI cases. Proportion of HPAI cases do not have any parent node, but have child nodes to lake irrigation, total irrigation, other irrigation, and river irrigation indicating indirect relationship between them. Variable expansion: LAKE_IRR: lake irrigation; TOT_IRR: total irrigation; TANK_IRR: tank irrigation; OTH_IRR: other irrigation; RIVER_IRR: river irrigation; W_FALL: water fall; CULT_WASTE: cultivable waste; No HH: number of household; TOT_P: total population; P_ILL: population illiterate; P_LIT: population literate; Other COM: other commodities (other than rice, rubber, and coir); AREA_NA_CU: area not available for cultivation; UN_IRR: unirrigated; TOT_EXP: total expenditure; TOT_INC: total income.

The six parent model was the best model based on log likelihood scores (higher the log likelihood score better is the model). Network graph of socio-economic variables and proportion of avian Influenza outbreaks with six parent limit is shown in Figure 3. The proportion HPAI cases do not have any parent node meaning that it is not directly related to any of the socio-economic variables considered in our study, but it has child nodes to lake irrigation, total irrigation, other irrigation, and river irrigation indicating indirect relationship.

### Semi-variance

**Figure 4.**
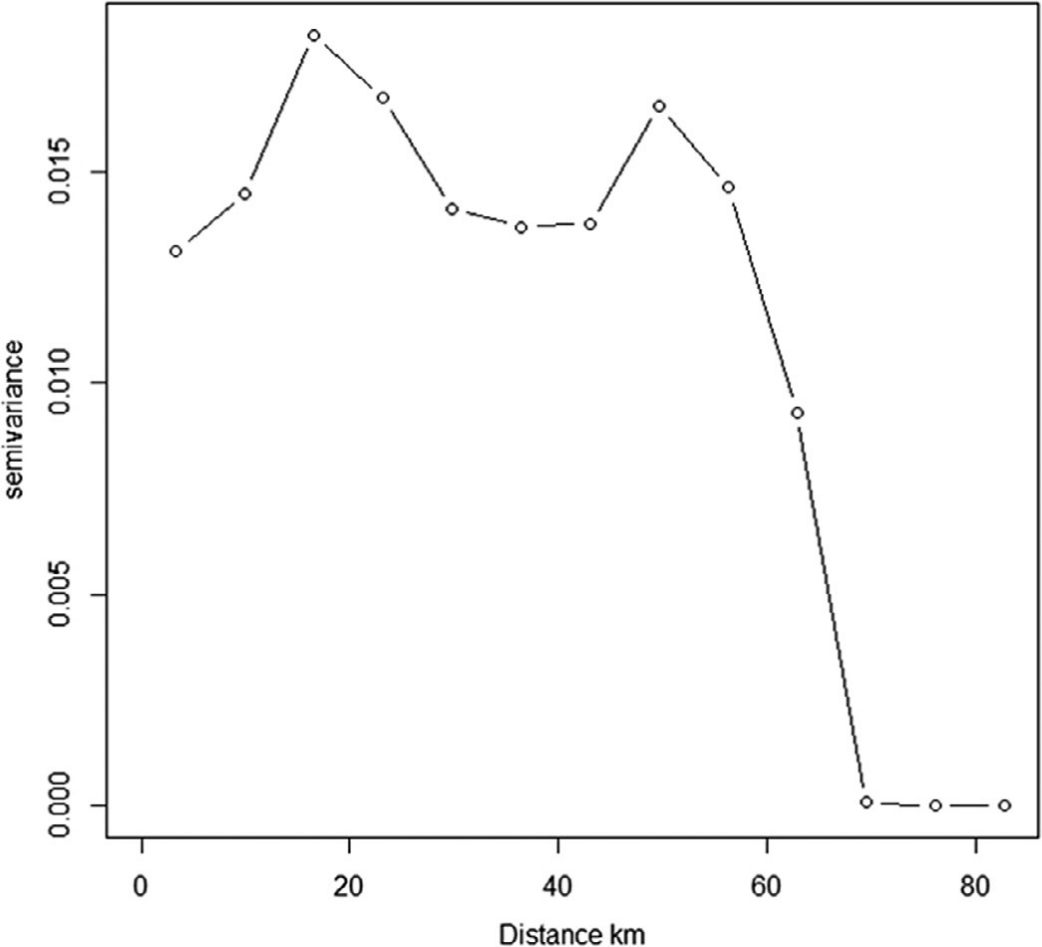
Semi-variogram analysis of proportion of H5N1 cases in duck population of Alappuzha and Kottayam districts. It shows that there is spatial dependency in the outbreaks upto 60 km.

The semi-variogram plot of the proportion of HPAI cases (Figure 4) showed that there was a spatial autocorrelation in the data. The spatial dependency in the occurrence of the outbreaks was detected up to 60 km and thereafter it declined.

### SPDE model results

**Figure 5.**
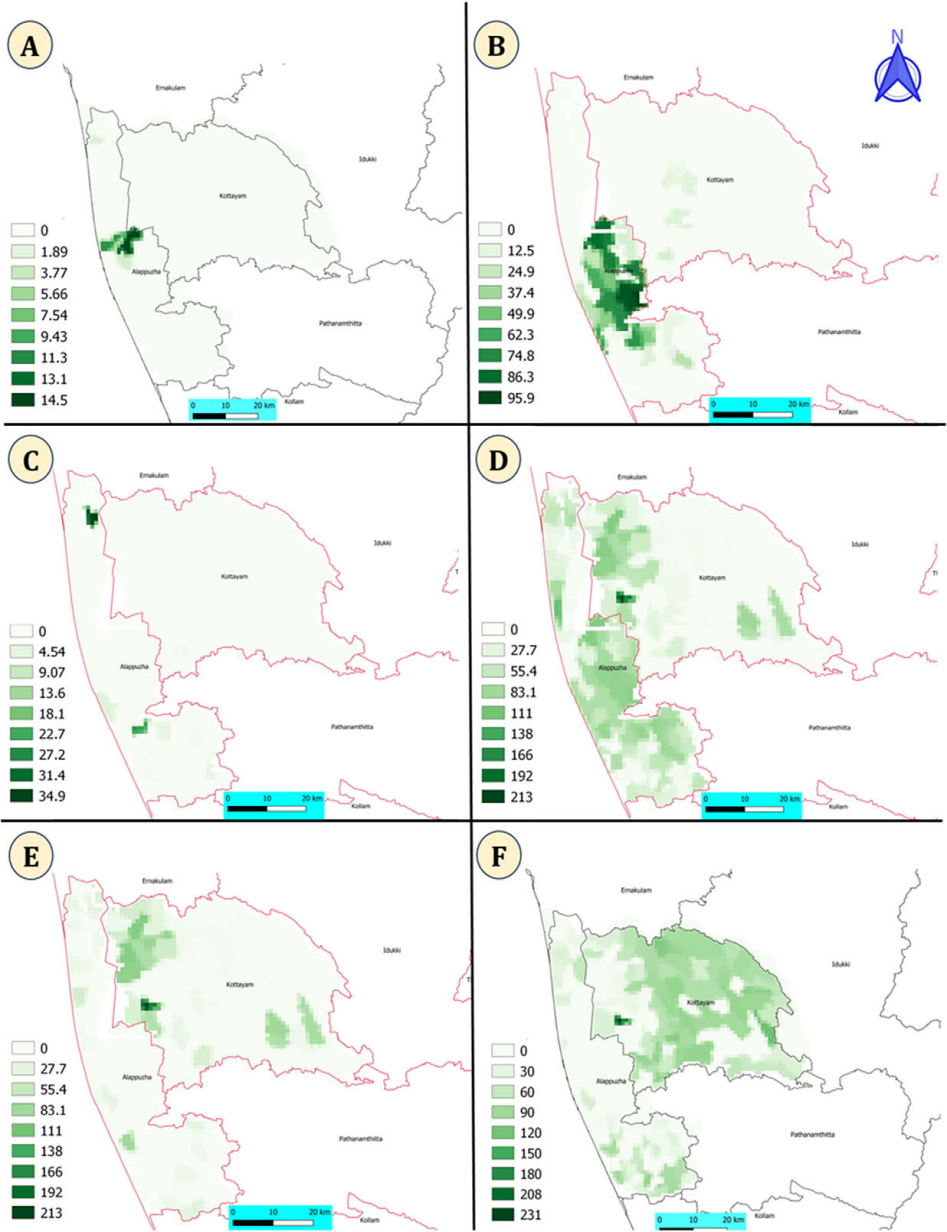
Predictor variables used in the Bayesian spatial SPDE model. (A): Lake irrigation; (B): river irrigation; (C): tank irrigation; (D): total irrigation; (E): other irrigated; (F): unirrigated. River and lake irrigation is mostly in the Alappuzha district.

The distributions of the irrigation variables in two districts are shown in Figure 5. Northern parts and few central areas of Kottayam district are irrigated with lake (Figure 5A). The river irrigation areas are more present in Southern parts of the Alappuzha district and few areas are river irrigated in Kottayam district (Figure 5B). There are few areas with tank irrigation in both the districts (Figure 5C). Majority of the areas in Alappuzha district and Northern areas of Kottayam district are irrigated (Figure 5D).

**Figure 6.**
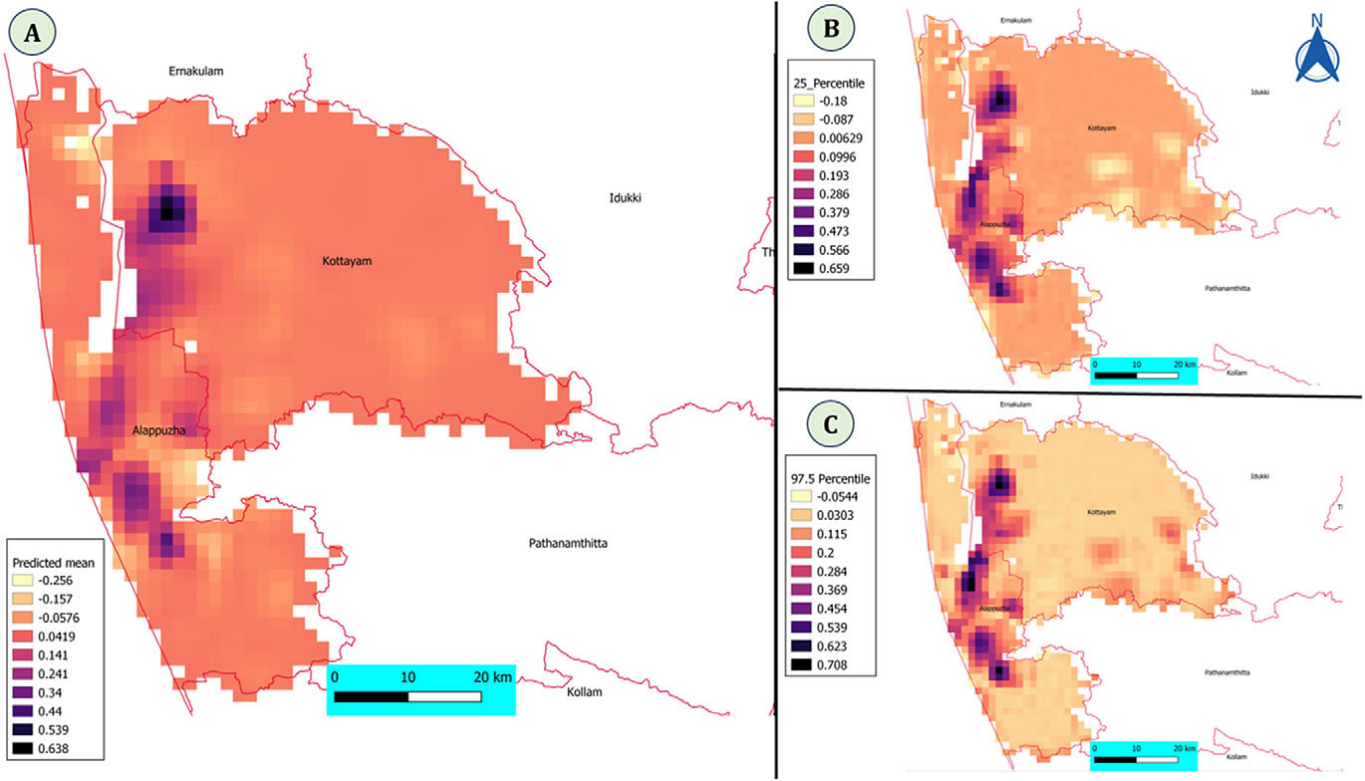
(A) Predicted mean using Bayesian spatial SPDE model with covariates. The risk of the disease is more in central parts of Alappuzha and North-Western parts of Kottayam district. (B and C) Model credible intervals (2.5 and 97.5 percentile) of the Bayesian spatial SPDE model with covariates.

The mesh constructed using constrained refine Delaunay triangulation is shown in Figure S1. The predictions using SPDE model with covariates show that the risk of disease is more in Southern parts of Alappuzha and Northern parts of Kottayam surrounding the wetland areas (Figure 6A). The uncertainty of the predictions (2.5 and 97.5 percentile) is shown in Figure 6B and C.

The SPDE model coefficients shows that various irrigation sources, such as lakes, rivers, tanks, and other irrigation as significant and positively associated with occurrence of HPAI outbreaks. (Table 1). The results of the model without covariate are shown in Table 2. The SPDE model without covariate is better compared to model with covariate based on DIC.

**Table 1.** Fixed and spatial effects with their mean, standard deviation, and credible interval of the Bayesian spatial SPDE model with covariates

S. no.	Variable	Mean (SD)	Credible interval
1.	Intercept	0.000 (0.016)	−0.032, 0.032
2.	**Lake irrigation**	**0.015** **(0.006)**	**0.003, 0.027**
3.	**Other irrigation**	**0.001** **(0.001)**	**0.000, 0.002**
4.	**River irrigation**	**0.002** **(0.001)**	**0.001, 0.004**
5.	**Tank irrigation**	**0.009** **(0.003)**	**0.004, 0.014**
6.	Total irrigation	0.000 (0.000)	−0.001, 0.001
7.	Unirrigated areas	0.000 (0.000)	0.000, 0.001
8.	Random effects		
9.	Theta 1	1.715 (0.163)	1.38, 2.018
10.	Theta 2	−0.797 (0.15)	−1.07, −0.485
11.	DIC	−1,062.37	
12.	Effective number of parameters	175.80	

Significant variables are highlighted in bold.

**Table 2.** Fixed and spatial effects with their mean, standard deviation, and credible interval of the Bayesian spatial SPDE model without covariate

Variable	Mean (SD)	Credible interval
Intercept	0.035 (0.013)	0.009, 0.06
Random effects		
Theta 1	1.73(0.045)	1.38, 1.805
Theta 2	−0.93 (0.043)	−1.01, −0.819
DIC	−1,037.85	
Effective number of parameters	220.65	

### Questionnaire data results

Attack rate of HPAI occurrence with different variables is shown in Table 3. The attack rate was maximum for duck farms that use more than five paddy fields for rearing of ducks (AR = 55%) followed by farms using one to four paddy fields (AR = 17.85%) and farms not using paddy fields (AR = 14.28%). The attack rate was higher (AR = 34.04%) in farms using paddy fields compared to farms not using paddy fields (R = 13.33%). The attack rate (AR = 28.84%) was higher in layer duck farms compared to ducks reared for meat purpose.

**Table 3.** Attack rate of different risk factors considered in the study

Risk factor	Attack rate (%)
Number of paddy fields	
Not using paddy fields	14.28
1–4 paddy fields	17.85
5 and above	55
Duck rearing paddy field	
No	13.33
Yes	34.04
Purpose of rearing	
Layer	28.84
For meat purpose	0
Both	60

Different model combinations also show that all variable models are the best models compared to all the other individual or combination models based on Akaike information criterion (AIC) (Table 4). The co-efficient of the logistic regression model is shown in Table 5. The number of paddy fields used by different duck farmers is positively associated with the occurrence of HPAI outbreaks in the two districts of Kerala.

**Table 4.** Different combinations of model with AIC

S. no.	Model	AIC
1.	All variable model	122.22
2.	Paddy field + rearing purpose	128.06
3.	Rearing purpose + number of paddy fields	122.01
4.	Number of paddy fields	126.11
5.	Paddy field	134.36

**Table 5.** Model co-efficient with different risk factors

Variable	Mean (SE)	*p*-Value
Intercept	−2.442 (0.7372)	0.000923
Rearing purpose 2	−17.75 (1600)	0.991146
Rearing purpose 3	0.007671 (0.6284)	0.990260
Paddy field	1.176 (0.922)	0.202004
**Number of paddy fields**	**0.3412** (0.1413)*	**0.015763**

Number of paddy fields is significant.

**Figure 7.**
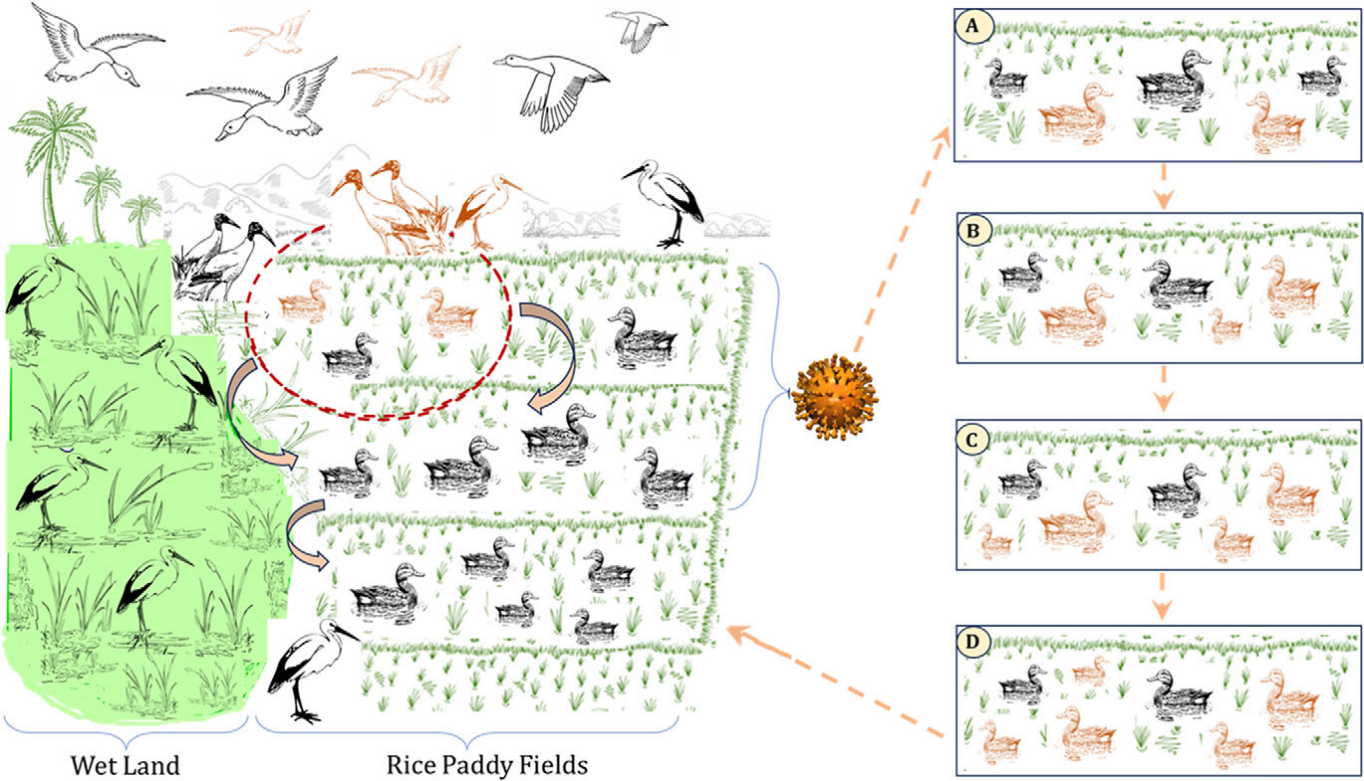
Unique duck rearing practice on multiple paddy fields in wetland areas of Alappuzha and Kottayam districts of Kerala. The shifting of paddy field is in both direction and on rotation basis.

The graphical representation of the unique duck rearing practices in this region is shown in Figure 7.

## Discussion

HPAI outbreaks are recurrently happening in two districts of Kerala state, India. The present study was taken up to identify the factors responsible for the spatial distribution and spread of HPAI in these two districts in Kerala. The BNM identified irrigation variables among the socio-economic factors indirectly associated with the proportion of cases. However, the SPDE model without covariates was the best model compared to the model with covariates showing the importance of spatial spread of the disease in the region. In addition, the number of rice paddy fields was identified as significant in local spread of the disease.

The majority of the outbreaks were reported from the wetland areas in the two districts of Kerala state, Alappuzha and Kottayam, that is, 36% of the total duck population in Kerala state (https://web.cdit.org/animalhusbandry/statistics/). The wetland areas are hubs for migratory birds. In one study, it was found that the proportion of flooded areas and expansion of rivers and streams were significant in indicating water-borne transmission of the H5N1 outbreaks in Thailand [34]. We found that the SPDE model without covariates was better in explaining the spatial variability in the outbreaks compared to the model with covariates. The spatial pattern might be due to the movement of migratory birds in the region or movement of people or ducks in the region or even water-borne transmission cannot be ruled out. The majority of the outbreaks were located within the irrigated areas. The indirect association of the irrigation variables identified in the BNM (Figure 3) and identification of the SPDE model without covariates as the best model shows the importance of spatial spread of disease, and it may be due to movement of people/ducks. The majority of the reported outbreaks are in wetland areas with rice paddy cultivation, and it may be possible that the first outbreak is happening due to migratory birds and spreading to other parts by movement of people/ducks. Similarly, in other studies, a Bayesian hierarchical model revealed the role of free-grazing ducks and rice cropping intensity along with anthropogenic factors in the spread of H5N1 outbreaks [35]. The spatial risk of H5N1 in chickens was also associated with the elevation, human population density, and rice cropping areas [36]. We used a BNM approach to select the variables that identify the direct and indirect relationships between variables and did not find total human population as directly or indirectly associated with HPAI outbreaks. Bayesian spatial modelling was used to identify differences in H5N1 and H5N6 outbreaks and account for spatial autocorrelation in Thailand and found that H5N1 outbreaks were explained by fixed effects compared to H5N6 outbreaks that were more spatially autocorrelated [37].

### Importance of irrigation variables

The irrigation variables (lake irrigation, river irrigation, tank irrigation, and other irrigation) were positively associated with the occurrence of outbreaks in the two districts in our study. In a another study, it was found that the risk of HPAI increases with a greater proportion of rice paddy fields, density of chickens, and ducks [38]. We used the proportion of the cases which accounted for the population of ducks in the analysis, and identification of irrigated areas in our analysis supports the finding from other studies. We did not use rice paddy cultivation data as it was not available at the panchayat level (admin level 4), but the wetland areas in this region are used for rice paddy cultivation [39]. Similar observations were found in Thailand wherein it was observed that wetlands used for both rice-paddy cultivation and free-grazing ducks were critical in the spread and persistence of H5N1 outbreaks [40]. In addition, it was found that duck abundance, human population, and rice cropping intensity were more important compared to chicken numbers [41]. An affected village <5 km from a river/stream was significant along with other factors in Romania [42].

### Risk factor identification using questionnaire

There was evidence of spatial dependence of the outbreaks identified in our SPDE model (Figure 4 and Table 1). In order to further investigate the factors responsible for spatial spread of the disease, we interviewed all the duck farmers in the region. In our questionnaire-based risk factor identification, we found that the number of rice paddy fields used by the duck farmers was significant and important in the spread of the disease in the region. There are many studies to show the importance of movement of ducks and free-ranging ducks in driving the outbreaks. It was found that the ducks that were scavenging to neighbouring houses and not confined overnight were at risk of developing H5 antibodies as observed in a longitudinal study [43]. Viral RNA prevalence was significantly higher in free-range duck flocks reared by illiterate farmers compared to ducks reared in households along with other risk factors in Bangladesh [44]. There was higher seroprevalence of H5 antibodies in ducks compared to chickens in-contact with ducks [45]. The presence of free-grazing ducks and simultaneous reports in wild birds along with other risk factors were significant in H5N1 outbreaks in Thailand [46]. In our study, we found that migratory birds are a likely source of infection and subsequent spatial spread of disease is happening due to unique duck rearing practices (Figure 7). Movement of duck population to feed on post-harvest paddy fields were responsible for the H5N1 outbreaks in Indonesia [47]. Similarly, purchase of live chickens from another backyard poultry farm was important and significant in transmission of H5N1 [48].

Although ducks are an important reservoir and are maintenance hosts of avian Influenza viruses, there is no virus shedding after 10 days as reported in an experimental study [49]. However, recent studies have shown that shedding can extend up to 14 days for H5N8 clade 2.3.4.4b virus [50]. This shows that the ducks may be acquiring the infection from migratory birds in every season and local transmission is dependent on the movement of infected ducks (Figure 7). Surveillance in duck production systems and prevention of intermingling with migratory birds in wetlands is recommended due to high prevalence and circulation of HPAI in ducks [51]. It has been noticed that H5 virus shedding occurs in 68.8% of farms in apparently health birds and ducks are the source of infection for chickens and potentially can be for humans [52].

## Limitation of the study

We have used reported outbreak data (year 2014–2022) in the region for our analyses, but there is always possibility of under-reporting. However, our spatial predictions in the region show that the risk of the disease is also restricted in and around the reported areas. We did not perform the parametric bootstrapping of the BNM model due to lack of computational resources. However, the BNM model did not identify any direct association between the socio-economic factors and occurrence of HPAI outbreaks, and hence, the BNM bootstrapping would have not affected the inference of our analysis, further supported by the SPDE model.

## Conclusion

Overall, we used robust statistical methods to identify the spatial pattern and risk factors in spread of the HPAI outbreaks for the years 2014–2022 in the study region. Our study shows that it is required to have intensive surveillance in the ducks of this region to prevent the spread and recurrence of HPAI outbreaks in the two districts of Kerala. The surveillance in ducks is not only important to prevent the outbreaks in the study region but also to prevent constant exposure of the virus to humans. In addition, appropriate intervention strategies have to be developed to convince duck farmers to modify the duck rearing practice in wetland and high-risk areas identified in our study. It is also important to safeguard the livelihood of the duck farmers in this high-risk region of wetland and hub of migratory birds.

## Supporting information

Chanda et al. supplementary material Chanda et al. supplementary material

## Data Availability

All the data used in the manuscript is available in open domain. All the statistical analysis details and R packages used are mentioned in the manuscript.

## Supplementary material

For supplementary material accompanying this paper visit http://doi.org/10.1017/S0950268824001882. click here to view supplementary material
