# Supplementary material for: Unique duck rearing practice in irrigated rice paddy fields driving recurrent H5N1 avian influenza outbreaks in two districts of Kerala, India
Source: Epidemiol Infect. 2025 Jan 7;153:e17. doi: 10.1017/S0950268824001882 (PMC11748019; doi:10.1017/S0950268824001882)
Supplement: Chanda et al. supplementary material [file S0950268824001882sup001.docx]

**Supplementary information**

**Unique duck rearing practice in irrigated rice paddy fields driving recurrent H5N1 Avian Influenza outbreaks in two districts of Kerala, India**

Mohammed Mudassar Chanda^1🖂^., Sathish Bhadravati Shivachandra^1^., Adhiraj Mishra^2^., Previn Punnoose^3^, Shaji Panikkassery^3^., Sanjay Devarajan Potti^3^., Vysakh Mohan^3^., Awadesh Prajapathi^1^., Revanaiah Yogisharadhya^4^ ., Divakar Hemadri^1^., Baldev Raj Gulati^1^., and Chakradhar Tosh^5^

^1^ICAR-National Institute of Veterinary Epidemiology and Disease Informatics (NIVEDI), Ramagondanahalli, Yelahanka, Bengaluru- 560 064, Karnataka, India

Phone: 0091-80-23093100 (Ext: 141)

Fax: 0091-80-23093222

^🖂^E-mail: [chandamudassar@gmail.com](mailto:chandamudassar@gmail.com)

^2^Department of Animal Husbandry and Dairying, New Delhi, India

^3^Kerala State Animal Husbandry department, Government of Kerala

^4^ICAR Krishi Vigyan Kendra (ICAR RC for NEH Region), Hailakhandi, Assam, India

^5^ICAR-National Institute of High Security Animal Diseases, Bhopal - 462 022, Madhya Pradesh, India.

*
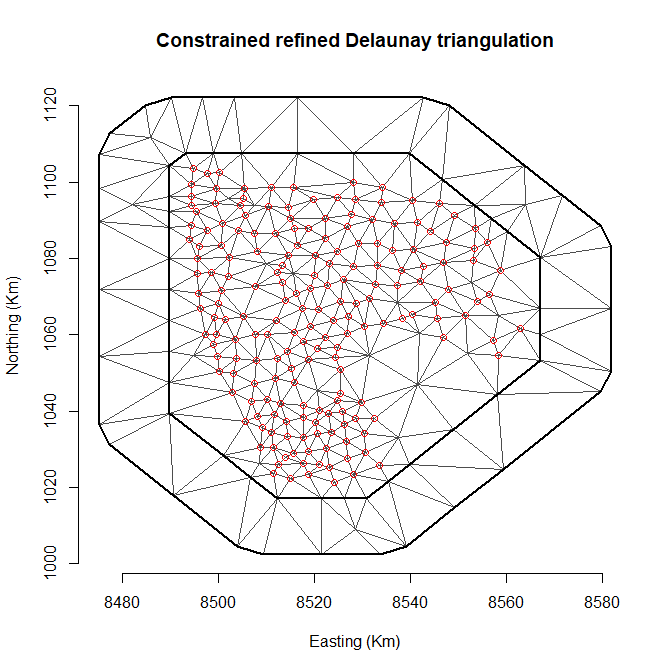
*

*Figure S1: Mesh construction using constrained refined Delaunay triangulation*

*Table S1: Questionnaire to identify risk factors for occurrence of Avian Influenza*

| **S.No.** | Name of the farmer | Contact number | Village | Whether Avian Influenza reported during the past three years (Years 2020, 2021, 2022)  (Mention as Yes/No) | Purpose of rearing (mention as A or B or C)  A. Layer B. Broiler C. Both | Duck rearing in Rice paddy field (Mention as Yes/No) | If Ducks are not reared in paddy field, then mention where they are reared  a.Pond b. River c. Near home d. Other (please mention) | How many Paddy fields are covered in a year? (please mention the number of paddy fields) | Can you provide details of vehicle used for transportation of Ducks? |
| --- | --- | --- | --- | --- | --- | --- | --- | --- | --- |
|  |  |  |  |  |  |  |  |  |  |
|  |  |  |  |  |  |  |  |  |  |
|  |  |  |  |  |  |  |  |  |  |
|  |  |  |  |  |  |  |  |  |  |
|  |  |  |  |  |  |  |  |  |  |
